# Concomitant Use of Dietary Supplements and Medicines in Patients due to Miscommunication with Physicians in Japan

**DOI:** 10.3390/nu7042947

**Published:** 2015-04-16

**Authors:** Tsuyoshi Chiba, Yoko Sato, Sachina Suzuki, Keizo Umegaki

**Affiliations:** Information Center, National Institute of Health and Nutrition, 1-23-1 Toyama, Shinjuku-ku, Tokyo 162-8636, Japan; E-Mails: satoyoko@nih.go.jp (Y.S.); sachina-s@nih.go.jp (S.S.); umegaki@nih.go.jp (K.U.)

**Keywords:** dietary supplements, patients, treatment of diseases, medication, adverse effects

## Abstract

We previously reported that some patients used dietary supplements with their medication without consulting with physicians. Dietary supplements and medicines may interact with each other when used concomitantly, resulting in health problems. An Internet survey was conducted on 2109 people who concomitantly took dietary supplements and medicines in order to address dietary supplement usage in people who regularly take medicines in Japan. A total of 1508 patients (two admitted patients and 1506 ambulatory patients) and 601 non-patients, who were not consulting with physicians, participated in this study. Purpose for dietary supplement use was different among ages. Dietary supplements were used to treat diseases in 4.0% of non-patients and 11.9% of patients, while 10.8% of patients used dietary supplements to treat the same diseases as their medication. However, 70.3% of patients did not declare dietary supplement use to their physicians or pharmacists because they considered the concomitant use of dietary supplements and medicines to be safe. A total of 8.4% of all subjects realized the potential for adverse effects associated with dietary supplements. The incidence of adverse events was higher in patients who used dietary supplements to treat their disease. Communication between patients and physicians is important for avoiding the adverse effects associated with the concomitant use of dietary supplements and medicines.

## 1. Introduction

The use of dietary supplements has recently increased worldwide. In the United States, 48.8% of the population used dietary supplements between 2007 and 2010 [1]. Dietary supplementation has also become more common in Japan, with 10.9% of the population using supplements in 2001 [2], 11.0% in males and 16.4% in females in 2003 [3] and 45.8% in older adults in 2008 [4]. Factors, such as sex, age, socioeconomic status and health-related characteristics, have been shown to affect the use of dietary supplements [1,5,6,7,8]. Furthermore, the purpose of dietary supplements has changed over time. Dietary supplements were previously used as a nutritional supplement, because malnutrition was a major health issue. However, dietary supplements are now used not only as nutritional supplements, but also in the prevention and treatment of diseases.

The current Japanese system for regulation of dietary supplements is called “Food with Health Claims” and is made up of two categories: (1) “Food with Nutrient Function Claims” for supplementation of vitamins or minerals; and (2) “Food for Specified Health Uses” for specific functions. However, there are a lot of products that people recognize as dietary supplements in the Japanese market other than “Food with Health Claims”. These products are not defined by law in Japan, even if they are in the form of capsules or tablets [9,10].

As dietary supplement use increases, associated health problems also increase. Adverse effects caused by dietary supplements, especially hepatotoxicity associated with herbal supplement use [11,12,13] or stroke and sudden death associated with adulterated supplement use [14,15], have been reported worldwide and have been attributed to two causes. The first cause is the use of low quality or illegal products that contain drug ingredients [16,17,18]; therefore, the Japanese government constantly surveys and checks these products on websites and retail stores. However, health problems that are caused by using adulterated dietary supplements are rare in Japan. The other cause, which is more important in Japan, is the inappropriate use of dietary supplements, including their excessive intake and the concomitant use of various dietary supplements and/or medicines. Japanese people do not appear to fully understand that dietary supplements are different from medicines, which has led to the use of dietary supplements to treat specific disease as medicines. In particular, inappropriate use of dietary supplements in patients may be associated with severe adverse effects.

We previously surveyed the use of dietary supplements by patients in Japan [19]. We found that some patients used dietary supplements concomitantly with medicines, but did not declare this to their physicians. Several reasons have contributed to this inappropriate use. Since a clear, official definition of dietary supplements does not currently exist in Japan, many dietary supplements claim to treat specific diseases, especially cancer, even though such claims are illegal. Previous studies reported that between 20% and 90% of cancer patients used dietary supplements as complementary and alternative medicines, not only in Japan [20], but also in other countries [21,22,23,24]. Furthermore, since dietary supplements are available as capsules or tablets, they have the appearance of medicines and, thus, are often considered to be as effective as medicines. The general public also does not understand the properties of dietary supplements. Physicians have previously expressed concerns regarding the use of dietary supplements by their patients due to the increased risk of dietary supplement-drug interactions [25]. Dietary supplements may interact with some drugs, as well as affect anesthesia and bleeding during surgery [26]. However, some physicians also do not understand the properties of dietary supplements [27,28].

Therefore, the concomitant use of dietary supplements and medicines may lead to dietary supplement-drug interactions and adverse effects. In the present study, an Internet survey was conducted by concomitant users of dietary supplements and medicines in order to clarify the risk of interactions among subjects in Japan.

## 2. Methods

### 2.1. Definition of Dietary Supplements

In Japan, the term dietary supplement has no definition in the law, and some dairy or soybean products are recognized as dietary supplements, even if they are in the form of common foods. Therefore, what dietary supplement means is different for each person. In this survey, we defined dietary supplements as those that had the form of capsules, tablets and powders and that subjects considered to have beneficial effects on their health.

### 2.2. Internet Survey

An Internet-based questionnaire was conducted by Macromill Inc. (Tokyo, Japan) on their research registrants between 18 July and 28 July 2014. Their registrants were more than 2 million in 2014. They could answer this questionnaire on a website and quit any time. This study was conducted with the approval of the Research Ethics Committee of the National Institute of Health and Nutrition. The questionnaire is shown in the supplementary file.

### 2.3. Preliminary Survey

To select concomitant users of dietary supplements and medicines, a preliminary survey was conducted on 270,083 subjects. Of this, 40,170 subjects completely answered the survey (response rate: 14.9%), and 7869 subjects (19.6%) were using dietary supplements and medicines concomitantly. The part of concomitant users were moved to the actual survey.

### 2.4. Actual Survey

The actual survey was conducted by 3129 subjects. The questionnaire included demographic characteristics (sex and age), information on their medical status, the purpose of supplementation (maintenance of health, improvements to health, for beauty or weight loss, prevention of diseases and treatment of diseases), the number and types of dietary supplements and medicines that they were taking and understanding of the beneficial and adverse effects. The questionnaire also asked whether subjects informed their physicians of their use of dietary supplements. Of the total subjects, 2109 subjects completely answered the survey (response rate: 67.4%). All subjects provided information on their medical status. Subjects who answered “I am an ambulatory patient (*n* = 1506)” or “I am an admitted patient (*n* = 2)” were categorized as patients, and subjects who answered “I am not consulting a doctor (*n* = 601)” were categorized as non-patients.

### 2.5. Statistical Analysis

Differences in demographic characteristics or the purposes among groups were tested using the χ^2^ test. A univariate analysis to determine the relationship between supplement use and various variables in patients and non-patients was also conducted using the χ^2^ test. Statistical analyses were performed using SPSS 18.0J for Windows (IBM Co. Armonk, NY). *p*-Values less than 0.05 were considered significant.

## 3. Results

### 3.1. Preliminary Survey

A preliminary survey was conducted on 40,170 people in order to identify those who concomitantly took dietary supplements and medicines. A total of 35.4% of all subjects were currently using dietary supplements; 43.7% took medicines regularly; and 19.6% (*n* = 7869) took dietary supplements and medicines concomitantly. On the other hand, 17.7% had ceased using dietary supplements for their medication. A part of the subjects taking concomitant medicines were allocated to the main survey in consideration of the population distribution (based on the sex, age and residence) in Japan.

### 3.2. Characteristics

The characteristics of all subjects (*n* = 2109) are shown in Table 1. The ratios of age and residence were adjusted by the population distribution in Japan. Among all subjects, 71.5% (*n* = 1508) were patients, including two admitted patients (Table 1), while the remainder (*n* = 601) were non-patients, even though they regularly took medicines. The ratio of patients to non-patients increased significantly with age.

**Table 1:** Characteristics of each group.

	Non-Patients	Patients	Total	*p*-Value
**Number of subjects, *n* (%)**	601 (28.5)	1508 (71.5)	2109 (100.0)	
**Sex, *n* (%)**				0.847
Male	302 (50.2)	749 (49.7)	1051 (49.8)	
Female	299 (49.8)	759 (50.3)	1058 (50.2)	
**Age, *n* (%)**				<0.001
20s	155 (25.8) ^a^	188 (12.5)	343 (16.3)	
30s	182 (30.3) ^a^	275 (18.2)	457 (21.7)	
40s	120 (20.0)	307 (20.4)	427 (20.2)	
50s	78 (13.0)	338 (22.4) ^a^	416 (19.7)	
60s	66 (11.0)	400 (26.5) ^a^	466 (22.1)	

Non-patients: subjects who answered “I am not consulting a doctor”, even though they regularly took medicine. *p*-Values were calculated using the χ^2^ test. ^a^ Correlation by an adjusted residual analysis (*p* < 0.05).

### 3.3. Prevalence of Dietary Supplements

Many subjects (53.7% of non-patients and 41.0% of patients) used some type of vitamin or mineral (including multi-vitamins, multi-minerals, or multi-vitamin/mineral products) (Table 2). Vitamins B and C and calcium, iron and zinc were the most commonly used vitamins and minerals, respectively. The preference for vitamin/mineral supplements was significantly greater in non-patients than in patients. Various kinds of non-vitamin, non-mineral dietary supplements were also used (Table 2). Blueberry/lutein products were the most frequently used by both non-patients and patients in this survey, followed by fish oil/n-3 PUFA and glucosamine/chondroitin. The preference for blueberry/lutein, fish oil/n-3 PUFA, glucosamine/chondroitin, black vinegar, garlic and sesamin products was significantly greater in patients than in non-patients. Furthermore, dietary supplements for weight loss, which were labeled as containing several ingredients, including *Coleus forskohlii*, *Gymnema sylvestre*, lactoferrin, α-lipoic acid or others, were also popular in Japan. A subgroup analysis showed that subjects who used dietary supplements to treat their diseases preferred to use non-vitamin/non-mineral supplements (14.8%, *n* = 236/1591 of non-vitamin/non-mineral supplements users), slightly more than vitamin and/or mineral supplements only (12.7%, *n* = 66/518 of vitamin and/or mineral supplements only users).

**Table 2:** Prevalence of the use of each type of dietary supplement.

Type of Dietary Supplement	Non-Patients	Patients	Total	*p*-Value
Number	601	1508	2109	
**Vitamin/Mineral**				
Multi-vitamins and minerals	24 (4.0)	59 (3.9)	83 (3.9)	1.000
Multi-vitamins	106 (17.6)	190 (12.6)	296 (14.0)	0.003
Multi-minerals	17 (2.8)	40 (2.7)	57 (2.7)	0.939
Each vitamin	161 (26.8)	322 (21.4)	483 (22.9)	0.009
Each mineral	111 (18.5)	196 (13.0)	307 (14.6)	0.002
Any type	323 (53.7)	619 (41.0)	942 (44.7)	<0.001
**Non-Vitamin, Non-Mineral (Top 10)**				
Blueberry/Lutein	60 (10.0)	205 (13.6)	265 (12.6)	0.029
Fish Oil/n-3 PUFA	37 (6.2)	174 (11.5)	211 (10.0)	<0.001
Glucosamine/chondroitin	30 (5.0)	163 (10.8)	193 (9.2)	<0.001
Collagen	31 (5.2)	79 (5.2)	110 (5.2)	1.000
Black vinegar	18 (3.0)	85 (5.6)	103 (4.9)	0.015
Garlic	13 (2.2)	79 (5.2)	92 (4.4)	0.003
Lactic bacterium	16 (2.7)	70 (4.6)	86 (4.1)	0.051
Sesamin	8 (1.3)	63 (4.2)	71 (3.4)	0.002
Curcuma longa	13 (2.2)	51 (3.4)	64 (3.0)	0.183
CoQ10	12 (2.0)	49 (3.2)	61 (2.9)	0.160
**Others**				
Weight loss supplements	31 (5.2)	83 (5.5)	114 (5.4)	0.644
St. John’s wort	5 (0.8)	0 (0.0)	5 (0.2)	-

Multiple answer *p*-values were calculated using the χ^2^ test.

### 3.4. Purpose of Dietary Supplement Use

The purpose of dietary supplement use is shown in Table 3 (medical status) and Table 4 (age). When medical status was compared (Table 3), no significant differences were observed in improvements to health, for beauty or weight loss or the prevention of diseases between both groups. However, significantly more non-patients than patients used dietary supplements to maintain health, whereas significantly more patients than non-patients used dietary supplements to treat diseases. When age was compared (Table 4), the use of dietary supplements to maintain health and prevent diseases was greater among older subjects than younger subjects. On the other hand, the use of dietary supplements to improve health and for beauty or weight loss was greater among younger subjects than older subjects. However, no significant difference was observed in the treatment of diseases among generations.

**Table 3:** Purpose for using dietary supplements (medical status).

	Yes	No	*p*-Value
**Maintenance of Health (%)**			
All subjects	54.1	45.9	
Non-patients	57.6	42.4	0.047
Patients	52.8	47.2	
**Improvements to Health (%)**			
All subjects	11.9	88.1	
Non-patients	13.3	86.7	0.206
Patients	11.3	88.7	
**For Beauty or Weight Loss (%)**			
All subjects	13.4	86.6	
Non-patients	15.0	85.0	0.203
Patients	12.8	87.2	
**Prevention of Diseases (%)**			
All subjects	8.0	92.0	
Non-patients	8.0	92.0	1.000
Patients	8.0	92.0	
**Treatment of Diseases (%)**			
All subjects	9.7	91.3	
Non-patients	4.0	96.0	<0.001
Patients	11.9	88.1	

*p*-Values were calculated using the χ^2^ test.

**Table 4:** Purpose for using dietary supplements (age).

	Yes	No	*p*-Value
**Maintenance of Health (%)**			<0.001
20s	41.7	58.3 ^a^	
30s	50.1	49.9 ^a^	
40s	55.0	45.0	
50s	59.6 ^a^	40.4	
60s	61.6 ^a^	38.4	
**Improvements to health (%)**			<0.001
20s	18.4 ^a^	81.6	
30s	13.6	86.4	
40s	12.4	87.6	
50s	9.1	90.9	
60s	7.5	92.5 ^a^	
**For beauty or weight loss (%)**			<0.001
20s	21.0 ^a^	79.0	
30s	20.4 ^a^	79.6	
40s	13.6	86.4	
50s	8.7	91.3 ^a^	
60s	5.2	94.8 ^a^	
**Prevention of diseases (%)**			<0.001
20s	5.2	94.8 ^a^	
30s	4.2	95.8 ^a^	
40s	7.7	92.3	
50s	9.6	90.4	
60s	12.4 ^a^	87.6	
**Treatment of diseases (%)**			0.372
20s	12.0	88.0	
30s	9.2	90.8	
40s	8.2	91.8	
50s	10.8	89.2	
60s	8.8	91.2	

*p*-Values were calculated using the χ^2^ test; ^a^ correlation by an adjusted residual analysis (*p* < 0.05).

### 3.5. Concomitant Use of Dietary Supplements and Medicines

As discussed above, 19.6% of participants (7869/40,170) in the preliminary survey took dietary supplements and medicines concomitantly, while the actual survey was only conducted on subjects who took dietary supplements and medicines concomitantly. Table 5 shows the number of subjects taking dietary supplements and medicines concomitantly. The most common pattern was one kind of dietary supplement and one kind of medicine (*n* = 440, 20.9%). However, 82 subjects (3.9%) took more than five dietary supplements and more than five medicines concomitantly.

**Table 5:** Number of dietary supplements and medicines used concomitantly.

Number of Medicines					
	1	2	3	4	≤5
Number of dietary supplements					
1	440	233	125	69	152
2	126	191	87	38	93
3	63	50	74	27	57
4	17	19	15	34	27
≤5	33	15	21	21	82

### 3.6. Declaration of Dietary Supplement Use to Physicians or Pharmacists

Only 25.7% of all subjects (16.0% of non-patients and 29.7% of patients) declared dietary supplement use to their physicians or pharmacists. In other words, more than 70% of patients used dietary supplements on their own without consulting with their physicians. Furthermore, neither of the admitted patients declared dietary supplement use to their attending physicians. Table 6 shows the reasons for not declaring dietary supplement use to physicians or pharmacists. Most subjects regarded dietary supplements as food and, therefore, safe and, as a consequence, did not consider interactions with their medication. In addition, “physicians or pharmacists never asked about dietary supplement use” was also a major reason for miscommunication between patients and physicians or pharmacists.

**Table 6:** Reasons for not declaring dietary supplement use to physicians or pharmacists.

Reasons	*n*
Dietary supplements are just food	653
There are no influences on medication (self-judgment)	509
There are no problems with using dietary supplements	369
Physicians or pharmacists never ask about dietary supplement use	360
Use dietary supplements only when needed	74
Physicians or pharmacists may deny dietary supplement use	31
Others	12

Multiple answers.

### 3.7. Beneficial or Adverse Effects due to the Use of Dietary Supplements

Only 41.1% of all subjects (40.8% of non-patients and 41.2% of patients) felt better by using dietary supplements. On the other hand, 8.4% of all subjects (8.2% of non-patients and 8.6% of patients) developed adverse effects from the use of dietary supplements, with the most common being diarrhea (32.7% of non-patients and 30.2% of patients), nausea and vomiting (14.3% and 25.6%), stomachache (22.4% and 12.4%), constipation (12.2% and 15.5%), fatigue (6.1% and 15.5%), headache (8.2% and 14.0%), interaction with medication (14.3% and 10.9%), rash and prurigo (6.1% and 10.1%), palpitations (2.0% and 8.5%), effects on health examination data (2.0% and 4.7%), and others. No significant difference was observed between non-patients and patients. A subgroup analysis showed that subjects who used any kind of non-vitamin/non-mineral supplement (8.7%) developed more adverse effects than those who only used vitamin and/or mineral supplements (7.7%). Furthermore, subjects who used dietary supplements to treat diseases (16.6%) had more adverse effects than those who used them for other purposes, such as the maintenance of health (8.4%), improvements to health (11.9%), for beauty or weight loss (10.3%) or the prevention of diseases (11.1%).

## 4. Discussion

We previously reported the inappropriate usage of dietary supplements by Japanese patients, some of whom used dietary supplements and medicines concomitantly without consulting with their physicians [19]. Some ingredients in dietary supplements have been shown to interact with drugs. Therefore, the concomitant use of dietary supplements and medicines by patients without the physician’s knowledge may lead to dietary supplement-drug interactions and adverse effects. In the present study, we investigated the concomitant usage of dietary supplements and medicines by patients in Japan.

The prevalence of dietary supplements has been surveyed, and vitamin/mineral supplements were revealed to be the most commonly used [4,29]. Consistent with a previously conducted survey, approximately half of our subjects used vitamin/mineral supplements. Deficiencies in vitamins/minerals cause health problems that can be prevented by vitamin/mineral supplements. The use of vitamin/mineral supplements was found to be increased in patients diagnosed with cancer [30]. In addition, vitamin/mineral supplements were shown to have beneficial effects in nutritionally deficient patients in China [31,32]. On the other hand, a cohort study in Japan [33] and a systematic review of vitamin/mineral supplements [34] showed that vitamin/mineral supplements did not affect the incidence of cardiovascular diseases and cancer or total mortality. It still remains unclear whether excessive amounts of vitamins/minerals are beneficial or harmful.

In addition to vitamin/mineral supplements, our subjects used various kinds of dietary supplements. The most popular ingredient in the dietary supplements used by our subjects was blueberry/lutein. Blueberry/lutein is also popular among non-medicated subjects in Japan, because it is considered beneficial for eye conditions, especially the prevention of cataracts and glaucoma. Blueberry/lutein products are marketed with such claims despite the lack of sufficient evidence, and the general public believes them in Japan. The second most popular ingredient was fish oil/n-3 PUFA, including eicosapentaenoic acid (EPA) and docosahexaenoic acid (DHA). EPA is also regulated as a drug ingredient for hyperlipidemia in Japan and has anti-platelet and anti-coagulant effects. Therefore, these dietary supplements are as effective as EPA-drugs, which are approved in Japan if they are of good quality. However, functional claims, such as for medicines, are not allowed to EPA-dietary supplements. Similar to EPA, chondroitin sulfate is regulated as a drug ingredient for rheumatoid arthritis in Japan. However, if efficacy is not claimed, chondroitin sulfate could be available as a dietary supplement. Most chondroitin supplements contain glucosamine, which is regulated as a drug ingredient for rheumatoid arthritis in Europe, but not in Japan, and are, thus, used by rheumatoid arthritis patients. A previous study reported that 60% of rheumatoid arthritis patients in Japan used dietary supplements, of which 40% are components of cartilage that contains chondroitin [35].

Dietary supplements have been shown to interact with drugs. The most well-known example is St. John’s wort (*Hypericum perforatum* L.). St. John’s wort contains hyperforin, which increases the expression of cytochrome P450 (CYP), especially CYP3A4, and affects drug metabolism in the liver [36]. In the present study, five non-patients used St. John’s wort, and none of them had declared this use to their physicians (pharmacists). Two subjects used sodium loxoprofen (a CYP2C9 substrate), with one reporting an interaction with their medication. Other subjects used paroxetine hydrochloride hydrate (a CYP2D6 substrate), vitamin B or folic acid with St. John’s wort. St. John’s wort does not interact with these medicines. If these subjects used St. John’s wort with this knowledge, it would not be considered problematic; however, they did not. Other herbs (e.g., black cohosh, *Coleus forskohlii*, echinacea, garlic, ginkgo, ginseng, green tea, kava and milk thistle) [37,38,39,40,41] and ingredients (e.g., catechins [42], curcuminoids [43], isoflavones [44], quercetin [45], polyphenols [46] and resveratrol [47]) also affect drug-metabolizing enzymes. In the present study, many subjects used these dietary supplements without consulting with their physicians.

Physicians need to be aware of the dietary supplements being used by their patients in order to avoid interactions between medication and dietary supplements. If there is insufficient evidence to warrant safety concerns, physicians need to advise their patients to cease the concomitant use of dietary supplements and medicines. However, 17% of all subjects answered that they did not discuss these supplements with their physicians or pharmacists, because they were never asked about it, which is consistent with previous findings [48]. This may have been associated with both patients’ and physicians’ insufficient recognition of the interactions between dietary supplements and medications, not only in Japan [43], but in other countries [49,50,51,52].

More subjects in the present study (8.4%) developed adverse effects due to the use of dietary supplements than in our previous study (3.3%) [19], even though most cases were not severe. No significant differences were observed in the incidence of adverse effects between non-patients and patients. This result suggests that the concomitant use of dietary supplements and medicines may increase the incidence of adverse effects. In this survey, we asked subjects which types of dietary supplements and medicines they were taking and found that most used several dietary supplements and medicines concomitantly. The possibility of dietary supplement-drug interactions is increased by the concomitant use of a larger number of dietary supplements and drugs. Therefore, we could not determine any relationship between dietary supplements and adverse effects.

There were some limitations to this study. This survey was conducted via the Internet, the utilization of which has recently expanded. The Ministry of Internal Affairs and Communications reported that the rate of Internet utilization was greater than 95% in those under 40s, 85% in those in their 50s, 63%–72% in their 60s and 49% in their 70s in 2012 in Japan. A gap may still exist between Internet users and non-users, especially in elderly people. Furthermore, Japanese people do not understand the difference between dietary supplements and medicines. A previous survey showed that 22% of the Japanese population did not understand the distinction between dietary supplements and medicines. In this study, some subjects inserted the name of a dietary supplement as a medicine name and *vice versa*. We also asked subjects for the names of the dietary supplements that they used, but a lot of subjects answered with just the main ingredient of the dietary supplement. Most products in Japan currently contain multiple ingredients. For example, if one product is being promoted as a glucosamine supplement, this product typically contains chondroitin, hyaluronic acid, collagen and other ingredients. Therefore, we were unable to clarify interactions between dietary supplements and medicines in this study.

## 5. Conclusions

It is important for physicians to ask patients about dietary supplement use and for patients to inform their physicians about these supplements if physicians do not ask about them. In addition, patients need more information on the dietary supplements that they use. Education about dietary supplements is important for patients in order to avoid the adverse effects associated with dietary supplements.

## Supplementary Files

Supplementary File 1

## References

[1] Bailey R.L., Gahche J.J., Miller P.E., Thomas P.R., Dwyer J.T. (2013). Why US adults use dietary supplements. JAMA Intern. Med..

[2] Ishihara J., Sobue T., Yamamoto S., Sasaki S., Akabane M., Tsugane S. (2001). Validity and reproducibility of a self-administered questionnaire to determine dietary supplement users among Japanese. Eur. J. Clin. Nutr..

[3] Ishihara J., Sobue T., Yamamoto S., Sasaki S., Tsugane S. (2003). Demographics, lifestyles, health characteristics, and dietary intake among dietary supplement users in Japan. Int. J. Epidemiol..

[4] Hirayama F., Lee A.H., Binns C.W., Watanabe F., Ogawa T. (2008). Dietary supplementation by older adults in Japan. Asia Pac. J. Clin. Nutr..

[5] Lyle B.J., Mares-Perlman J.A., Klein B.E., Klein R., Greger J.L. (1998). Supplement users differ from nonusers in demographic, lifestyle, dietary and health characteristics. J. Nutr..

[6] Messerer M., Johansson S.E., Wolk A. (2001). Sociodemographic and health behaviour factors among dietary supplement and natural remedy users. Eur. J. Clin. Nutr..

[7] Block G., Jensen C.D., Norkus E.P., Dalvi T.B., Wong L.G., McManus J.F., Hudes M.L. (2007). Usage patterns, health, and nutritional status of long-term multiple dietary supplement users: A cross-sectional study. Nutr. J..

[8] Wu C.H., Wang C.C., Kennedy J. (2011). Changes in herb and dietary supplement use in the U.S. adult population: A comparison of the 2002 and 2007 national health interview surveys. Clin. Ther..

[9] Yamada K., Sato-Mito N., Nagata J., Umegaki K. (2008). Health claim evidence requirements in Japan. J. Nutr..

[10] Ohama H., Ikeda H., Moriyama H. (2006). Health foods and foods with health claims in Japan. Toxicology.

[11] Teschke R., Wolff A., Frenzel C., Schulze J., Eickhoff A. (2012). Herbal hepatotoxicity: A tabular compilation of reported cases. Liver Int..

[12] Bjelakovic G., Gluud L.L., Nikolova D., Bjelakovic M., Nagorni A., Gluud C. (2011). Antioxidant supplements for liver diseases. Cochrane Database Syst. Rev..

[13] Pilkington K., Boshnakova A. (2012). Complementary medicine and safety: A systematic investigation of design and reporting of systematic reviews. Complement Ther. Med..

[14] Young C., Oladipo O., Frasier S., Putko R., Chronister S., Marovich M. (2012). Hemorrhagic stroke in young healthy male following use of sports supplement Jack3d. Mil. Med..

[15] Archer J.R., Dargan P.I., Lostia A.M., van der Walt J., Henderson K., Drake N., Sharma S., Wood D.M., Walker C.J., Kicman A.T. (2015). Running an unknown risk: A marathon death associated with the use of 1,3-dimethylamylamine (DMAA). Drug Test Anal..

[16] Pawar R.S., Tamta H., Ma J., Krynitsky A.J., Grundel E., Wamer W.G., Rader J.I. (2013). Updates on chemical and biological research on botanical ingredients in dietary supplements. Anal. Bioanal. Chem..

[17] Cohen P.A. (2012). DMAA as a dietary supplement ingredient. Arch. Intern. Med..

[18] Cohen P.A., Maller G., DeSouza R., Neal-Kababick J. (2014). Presence of banned drugs in dietary supplements following FDA recalls. JAMA.

[19] Chiba T., Sato Y., Nakanishi T., Yokotani K., Suzuki S., Umegaki K. (2014). Inappropriate usage of dietary supplements in patients by miscommunication with physicians in Japan. Nutrients.

[20] Hyodo I., Amano N., Eguchi K., Narabayashi M., Imanishi J., Hirai M., Nakano T., Takashima S. (2005). Nationwide survey on complementary and alternative medicine in cancer patients in Japan. J. Clin. Oncol..

[21] Klafke N., Eliott J.A., Wittert G.A., Olver I.N. (2012). Prevalence and predictors of complementary and alternative medicine (CAM) use by men in Australian cancer outpatient services. Ann. Oncol..

[22] Paul M., Davey B., Senf B., Stoll C., Munstedt K., Mucke R., Micke O., Prott F.J., Buentzel J., Hubner J. (2013). Patients with advanced cancer and their usage of complementary and alternative medicine. J. Cancer Res. Clin. Oncol..

[23] Singendonk M., Kaspers G.J., Naafs-Wilstra M., Meeteren A.S., Loeffen J., Vlieger A. (2013). High prevalence of complementary and alternative medicine use in the dutch pediatric oncology population: A multicenter survey. Eur. J. Pediatr..

[24] Frenkel M., Sierpina V. (2014). The use of dietary supplements in oncology. Curr. Oncol. Rep..

[25] Sadovsky R., Collins N., Tighe A.P., Brunton S.A., Safeer R. (2008). Patient use of dietary supplements: A clinician’s perspective. Curr. Med. Res. Opin..

[26] Messina B.A. (2006). Herbal supplements: Facts and myths—Talking to your patients about herbal supplements. J. Perianesth. Nurs..

[27] Kemper K.J., Gardiner P., Gobble J., Woods C. (2006). Expertise about herbs and dietary supplements among diverse health professionals. BMC Complement. Altern. Med..

[28] Ashar B.H., Rice T.N., Sisson S.D. (2007). Physicians’ understanding of the regulation of dietary supplements. Arch. Intern. Med..

[29] Park S.Y., Murphy S.P., Martin C.L., Kolonel L.N. (2008). Nutrient intake from multivitamin/mineral supplements is similar among users from five ethnic groups: The multiethnic cohort study. J. Am. Diet. Assoc..

[30] Velicer C.M., Ulrich C.M. (2008). Vitamin and mineral supplement use among US adults after cancer diagnosis: A systematic review. J. Clin. Oncol..

[31] Li J.Y., Taylor P.R., Li B., Dawsey S., Wang G.Q., Ershow A.G., Guo W., Liu S.F., Yang C.S., Shen Q. (1993). Nutrition intervention trials in Linxian, China: Multiple vitamin/mineral supplementation, cancer incidence, and disease-specific mortality among adults with esophageal dysplasia. J. Natl. Cancer Inst..

[32] Qiao Y.L., Dawsey S.M., Kamangar F., Fan J.H., Abnet C.C., Sun X.D., Johnson L.L., Gail M.H., Dong Z.W., Yu B. (2009). Total and cancer mortality after supplementation with vitamins and minerals: Follow-up of the Linxian General Population Nutrition Intervention Trial. J. Natl. Cancer Inst..

[33] Hara A., Sasazuki S., Inoue M., Shimazu T., Iwasaki M., Sawada N., Yamaji T., Ishihara J., Iso H., Tsugane S. (2011). Use of vitamin supplements and risk of total cancer and cardiovascular disease among the Japanese general population: A population-based survey. BMC Public Health.

[34] Alexander D.D., Weed D.L., Chang E.T., Miller P.E., Mohamed M.A., Elkayam L. (2013). A systematic review of multivitamin-multimineral use and cardiovascular disease and cancer incidence and total mortality. J. Am. Coll. Nutr..

[35] Ikuyama S., Imamura-Takase E., Tokunaga S., Oribe M., Nishimura J. (2009). Sixty percent of patients with rheumatoid arthritis in Japan have used dietary supplements or health foods. Mod. Rheumatol..

[36] Madabushi R., Frank B., Drewelow B., Derendorf H., Butterweck V. (2006). Hyperforin in St. John’s wort drug interactions. Eur. J. Clin. Pharmacol..

[37] Izzo A.A., Ernst E. (2009). Interactions between herbal medicines and prescribed drugs: An updated systematic review. Drugs.

[38] Shord S.S., Shah K., Lukose A. (2009). Drug-botanical interactions: A review of the laboratory, animal, and human data for 8 common botanicals. Integr. Cancer Ther..

[39] Hermann R., von Richter O. (2012). Clinical evidence of herbal drugs as perpetrators of pharmacokinetic drug interactions. Planta Med..

[40] Yokotani K., Chiba T., Sato Y., Taki Y., Yamada S., Shinozuka K., Murata M., Umegaki K. (2012). Hepatic cytochrome p450 mediates interaction between warfarin and *Coleus forskohlii* extract *in vivo* and *in vitro*. J. Pharm. Pharmacol..

[41] Virgona N., Yokotani K., Yamazaki Y., Shimura F., Chiba T., Taki Y., Yamada S., Shinozuka K., Murata M., Umegaki K. (2012). *Coleus forskohlii* extract induces hepatic cytochrome p450 enzymes in mice. Food Chem. Toxicol..

[42] Muto S., Fujita K., Yamazaki Y., Kamataki T. (2001). Inhibition by green tea catechins of metabolic activation of procarcinogens by human cytochrome p450. Mutat Res..

[43] Bamba Y., Yun Y.S., Kunugi A., Inoue H. (2011). Compounds isolated from *Curcuma aromatica* Salisb. Inhibit human p450 enzymes. J. Nat. Med..

[44] Nakajima M., Itoh M., Yamanaka H., Fukami T., Tokudome S., Yamamoto Y., Yamamoto H., Yokoi T. (2006). Isoflavones inhibit nicotine *C*-oxidation catalyzed by human CYP2A6. J. Clin. Pharmacol..

[45] Chen Y., Xiao P., Ou-Yang D.S., Fan L., Guo D., Wang Y.N., Han Y., Tu J.H., Zhou G., Huang Y.F. (2009). Simultaneous action of the flavonoid quercetin on cytochrome p450 (CYP) 1A2, CYP2A6, *N*-acetyltransferase and xanthine oxidase activity in healthy volunteers. Clin. Exp. Pharmacol. Physiol..

[46] Kimura Y., Ito H., Ohnishi R., Hatano T. (2010). Inhibitory effects of polyphenols on human cytochrome p450 3A4 and 2C9 activity. Food Chem. Toxicol..

[47] Detampel P., Beck M., Krahenbuhl S., Huwyler J. (2012). Drug interaction potential of resveratrol. Drug Metab. Rev..

[48] Mehta D.H., Gardiner P.M., Phillips R.S., McCarthy E.P. (2008). Herbal and dietary supplement disclosure to health care providers by individuals with chronic conditions. J. Altern. Complement. Med..

[49] Samuels N., Zisk-Rony R.Y., Zevin S., Becker E.L., Yinnon A.M., Oberbaum M. (2012). Use of non-vitamin, non-mineral (NVNM) supplements by hospitalized internal medicine patients and doctor-patient communication. Patient Educ. Couns..

[50] Binkowska-Bury M., Januszewicz P., Wolan M., Sobolewski M., Krauze M., Fijalek Z.E. (2013). Counterfeit medicines in Poland: Opinions of primary healthcare physicians, nurses and lay persons. J. Clin. Nurs..

[51] Cellini M., Attipoe S., Seales P., Gray R., Ward A., Stephens M., Deuster P.A. (2013). Dietary supplements: Physician knowledge and adverse event reporting. Med. Sci. Sports Exerc..

[52] Gardiner P., Sadikova E., Filippelli A.C., White L.F., Jack B.W. (2015). Medical reconciliation of dietary supplements: Don’t ask, don’t tell. Patient Educ. Couns..

