# Supplementary material for: Concomitant Use of Dietary Supplements and Medicines in Patients due to Miscommunication with Physicians in Japan
Source: Nutrients. 2015 Apr 16;7(4):2947–60. doi: 10.3390/nu7042947 (PMC4425182; doi:10.3390/nu7042947)
Supplement: Supplementary File 1 [file nutrients-07-02947-s001.docx]

Supplementary information

Preliminary Survey

Q1: At present, are you regularly taking any medicine?

A1: Yes, I am taking prescribed medicines.

A2: Yes, I am taking over-the-counter medicines.

A3: No, I am not.

Q2: At present, are you using any dietary supplements which formed as capsules, tablets, or powdered.

A1: Yes, I am using dietary supplements.

A2: No. I used to use dietary supplements, but I quitted (by myself).

A3: No. I used to use dietary supplements, but I quitted (by physicians advise).

A4: No. I used to use dietary supplements, but I quitted (by other reasons).

A5: No, I have never used.

A part of people who answered “A1 or A2 in Q1” and “A1 in Q2” moved to actual survey.

Actual Survey

Q1: What is your medical situation?

A1: I am an ambulatory patient.

A2: I am an admitted patient.

A3: I am not consulting a doctor.

Q2: How many medicines are you regularly taking?

Q3: Please write down names of medicines that you are taking.

Q4: How many dietary supplements are you regularly using?

Q5: Please write down names of dietary supplements that you are using.

Q6: Which purpose are you using dietary supplements for?

A1: maintain health

A2: beauty or weight loss

A3: improve health condition

A4: prevent diseases

A5: treat diseases (same disease with medication)

A6: treat diseases (other disease with medication)

A7: I am using it before starting medication.

A8: others

Q7: What is the motivation to start dietary supplement use?

A1: TV commercials

A2: newspapers, magazines, or advertisements

A3: internet

A4: at the store

A5: concerning health

A6: diagnosed of disease

A7: advice from physicians, pharmacists, or dieticians

A8: recommend in health guidance

A9: advice at drug store

A10: advice from family or friends

A11: others

Q8: Do you declare dietary supplements use to physicians or pharmacists?

A1: Yes, I do.

A2: No, I don’t.

Q8-2: What is the reason why you don’t declare?

A1: Dietary supplements are just food.

A2: Dietary supplements are not associated to disease or medicines that I am taking.

A3: I have never experienced any problems by using dietary supplements

A4: I use them every once in a while.

A5: I have never been asked by physicians or pharmacists.

A6: Physicians or pharmacists may deny using them.

A7: others

A8: There are no specific reasons.

Q9: Could you experience beneficial effects by using dietary supplements?

A1: Yes, I do.

A2: No, I don’t.

A3: I don’t know.

Q10: Have you ever experienced any adverse effects by using dietary supplements?

A1: No, I have never.

A2: nausea, vomit

A3: headache

A4: stomachache

A5: diarrhea

A6: constipation

A7: rash, itch

A8: fatigue

A9: palpitation

A10: affect health examination data (ex. fasting blood glucose, lipids level, or blood pressure)

A11: affect medication (increase or decrease efficacy of medicines)

A12: others
